# Adsorption behaviour of hydrogarnet for humic acid

**DOI:** 10.1098/rsos.172023

**Published:** 2018-04-11

**Authors:** Hirotaka Maeda, Yuichi Kurosaki, Masanobu Nakayama, Emile Hideki Ishida, Toshihiro Kasuga

**Affiliations:** 1 Department of Life Science and Applied Chemistry, Nagoya Institute of Technology, Gokiso-cho, Showa-ku, Nagoya 466-8555, Japan; 2 Graduate School of Environmental Studies, Tohoku University, 6-6-20 Aoba, Aramaki, Aoba-ku, Sendai, 980-8579, Japan

**Keywords:** hydrogarnet, humic acid, adsorption, hydroxyl groups

## Abstract

Discharge of humic acid (HA) in aqueous environments is a key health and aesthetic issue. The present work investigates the use of hydrogarnet as a novel adsorbent for HA. Hydrogarnet was hydrothermally synthesized with different solvents to control the chemical composition. Hydrogarnet with three types of chemical compositions had better adsorption properties for HA than hydrogarnet with a single chemical composition. Controlling the chemical composition of hydrogarnet increased the number of hydroxyl groups and the overall binding energy of the system, leading to changes in the zeta potential. The enhancement of these adsorption properties is related to the increased numbers of hydroxyl groups on the surface and their diverse binding energies.

## 1. Introduction

Humic acid (HA) is a ubiquitous natural organic polyelectrolyte formed by the breakdown of animal and vegetable matter in aqueous environments. The existence of large amounts of HA in water causes a yellowish to brown colour, and chlorine used during water purification reacts with HA to form carcinogenic material [1]. Discharge of HA in water is, therefore, a major environmental and health concern.

A number of methods, including membrane separation and coagulation techniques, have been reported to remove HA from effluents [2,3]. HA can also be removed from aqueous solution by adsorption using micropores and functionalized substrate filters, which act as adsorption sites. In the case of activated carbon, the pores can become blocked by adsorption of HA [4]; therefore, carbon filters with mesopores were prepared to overcome this problem [5]. On the other hand, kaolinite features higher adsorption for HA than montmorillinite, although they have similar surface functional groups (Al-OH and Si-OH) [6]. Adsorption of HA on Mg/Al-layered double hydroxides occurs by a ligand exchange reaction with the surface groups [7]. As such, we believe that the binding energies of the surface functional groups play an important role in the HA adsorption properties.

HA contains various organic functional groups such as hydroxyl, carboxylic and phenolic groups, leading to the existence of reaction sites with different potential energies. It is difficult to develop overall removal of HA from aqueous environments because of its complex structure. Increasing the number of interactions between HA and the material surface is a key factor for improving the adsorption properties in HA removal systems. To manipulate various adsorption sites for HA at the material surface, the hydroxyl groups present would need to have a diverse range of binding energies. We expect that these groups would be easy to tune as sites for reaction with HA. The hydrogarnet series Ca_3_Al_2_(SiO_4_)_3-*x*_(OH)_4*x*_ (0 ≤ *x* ≤ 3) is a solid solution consisting of grossular (*x* = 0) and katoite (*x* = 3). The increased substitution of (SiO_4_)^4−^ in this structure causes a decrease in the lattice constants, leading to disorder of the cation-centred polyhedral [8]. The Wulff shapes, which simulate the morphology with different chemical compositions of grossular and katoite calculated by *ab initio* density functional theory, indicated a different trend of the relative ratio of crystal planes present based on chemical composition [9]. This implies that the chemical composition of hydrogarnet would influence the number and binding energies of the hydroxyl groups present. In this work, HA adsorption by hydrogarnet with different chemical compositions was investigated to better elucidate the effect of hydroxyl groups on the overall adsorption behaviour.

## 2. Material and methods

We have successfully controlled the chemical compositions of hydrogarnet using hydrothermal conditions [9]. A slurry consisting of silica gel, calcium hydroxide, γ-alumina and dilute potassium hydroxide solution (0.05* *M) as solvent with a 1 : 10 solid : solvent ratio was hydrothermally reacted at 150°C for 6 h to prepare hydrogarnet with three types of chemical composition (denoted as KOH-Hg). The molar ratio of Ca : Al : Si was 3 : 2 : 1 in the slurry. Hydrogarnet with a single chemical composition was prepared as a control material under the same hydrothermal reaction using a slurry consisting of silica gel, calcium hydroxide, γ-alumina and distilled water as a solvent (denoted as DW-Hg).

The crystalline phase of the samples was analysed by X-ray diffraction (XRD) with Cu-K*α* radiation using corundum as an internal standard. X-ray photoelectron spectra (XPS) of Al_2p_ and Si_2p_ in the samples were measured at 10^–7^ torr using monochromatic Al-K*_α_* irradiation. The binding energy was normalized to the C_1s_ peak at 284.8 eV. The Al_2p_ spectra were deconvoluted into two peaks at 74.4 eV due to Al-OH [10] and 74.8 eV due to Al–O–Si [11]. Si_2p_ spectra were deconvoluted into two peaks at 101.8 eV due to Si-OH [12] and 102.3 eV due to Si–O–Al [13]. All spectra were fitted with a Voigt function (Gaussian : Lorentz = 70 : 30) [14].

The HA adsorption capacity of the samples was evaluated using 2.5 g l^–1^ adsorbent. HA solution was prepared using a method first reported by Moriguchi *et al*. [15]. In brief, HA was dissolved in a diluted NaOH solution, and the solution was filtered using a membrane filter with 0.45 µm pores. The solution was added to a diluted HCl solution for precipitation and centrifuged to obtain HA. After dissolution of the resultant HA in diluted NaOH, diluted HCl was added to shift the pH of the HA solution to 7. The samples were soaked in HA solution for 24 h, and the supernatant obtained by centrifugation of the slurry solution was analysed using UV/Vis spectroscopy by monitoring the change in absorbance at 400 nm. Blank samples without adsorbent were measured for each adsorption test as a reference.

## 3. Results and discussion

Peaks corresponding to hydrogarnet were present in the XRD patterns of both samples. The chemical composition of hydrogarnet was determined from the linear equation prepared by plotting the *x*-value in their series against the d-spacing of the major peak (420) at approximately 32° of the known patterns in the International Centre for Diffraction Data database [16]. The chemical composition of DW-Hg was Ca_3_Al_2_(SiO_4_)_0.42_(OH)_10.32_. KOH-Hg featured three types of chemical composition, these being Ca_3_Al_2_(SiO_4_)_1.31_(OH)_6.76_, Ca_3_Al_2_(SiO_4_)_0.66_(OH)_9.37_ and Ca_3_Al_2_(SiO_4_)_0.31_(OH)_10.76_. The semi-quantitative XRD analysis showed that the relative ratios for Ca_3_Al_2_(SiO_4_)_1.31_(OH)_6.76_, Ca_3_Al_2_(SiO_4_)_0.66_(OH)_9.37_ and Ca_3_Al_2_(SiO_4_)_0.31_(OH)_10.76_ were determined to be 19%, 44% and 37%, respectively [9]. The hydrogarnet ratio in both samples was almost the same. Both samples showed a similar specific surface area (35 m^2^ g^–1^) based on nitrogen gas sorption analysis.

**Figure 1.**
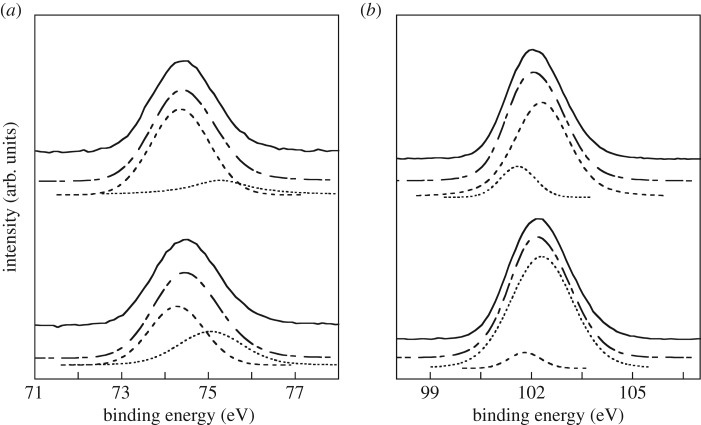
(*a*) Al_2p_ and (*b*) Si_2p_ XPS spectra. Solid, dashed and dash-dotted lines represent original, fitted and the sum of fitted spectra, respectively.

Figure 1 shows Al_2p_ and Si_2p_ XPS spectra of the samples. The number of hydroxyl groups arising from Al-OH and Si-OH on the material surface was evaluated by the relative portion of the integrated XPS peaks in each XPS spectrum. The percentages of Al-OH for DW-Hg and KOH-Hg were determined to be approximately 70% and 90%, respectively. The percentages of Si-OH for DW-Hg and KOH-Hg were determined to be 10% and 25%, respectively. The full-width at half maxima (FWHM) for both hydroxyl groups of KOH-Hg in Al_2p_ and Si_2p_ XPS spectra were larger than those of DW-Hg. The decreased substitution of (SiO_4_)^4−^ in the hydrogarnet by controlling the chemical compositions causes change in the crystal structure. This is supposed to induce various crystal planes present, resulting in an increased number of hydroxyl groups and a wider range of their binding energies. The zeta potentials at pH = 7 of DW-Hg and KOH-Hg were determined by a laser Doppler velocimeter to be approximately 0 mV and 20 mV, respectively. This is supposed to be caused by the differences in their hydroxyl groups.

**Figure 2.**
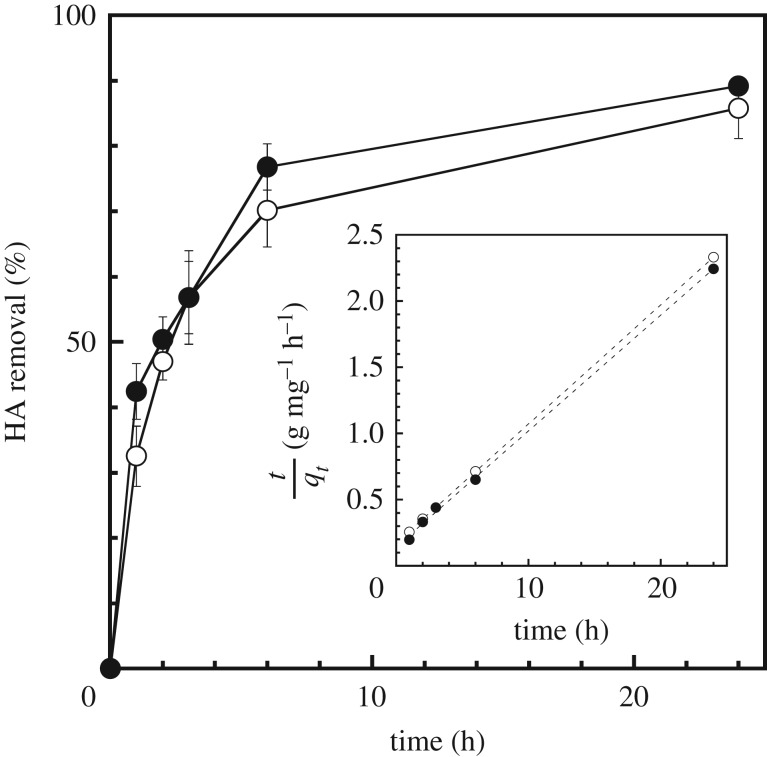
HA adsorption kinetics of DW-Hg (open circles) and KOH-Hg (filled circles). The inset shows data fitted to a pseudo-second-order model.

To demonstrate the HA adsorption properties of samples, experimental adsorption kinetics were determined using an initial concentration of 30 ppm, as shown in figure 2. The kinetic curves of HA adsorption based on two different kinetic models were investigated to clarify their effect on the kinetic properties using the following equations for pseudo-first-order and pseudo-second-order kinetics:

3.1 $$\ln\;(q_{e}-q_{t})=\ln\;(q_{e})-k_{1}t$$

and

3.2 $$\frac{t}{q_{t}}=\frac{1}{k_{2}q_{e}^{2}}+\frac{1}{q_{e}}t,$$

where *q*_e_ and *q_t_* (mg g^−1^) indicate the amount of HA adsorbed at equilibrium and adsorption time, respectively, and *k*_1_ (1/h) and *k*_2_ (g/mg h^−1^) are the rate constants of adsorption. The models for pseudo-first-order and pseudo-second-order equations described more than 0.98 and 0.99 of the correlation coefficients, respectively. Comparing the correlation coefficients of both models shows that the adsorption mechanism is a better fit to the pseudo-second-order equation. *q*_e_ and *k*_2_ values were calculated from the slope and intercept of the linear plots, shown in the inset of figure 2. The KOH-Hg sample had a slightly higher maximum adsorption capacity (11.4 mg g^−1^) compared with DW-Hg (11.1 mg g^−1^). Zeolite A-3 and molecular sieve 5A indicated 30–60% removal of HA [17]. It was reported that 40% removal of HA is achieved on kaolinite [18]. Hydrogarnet shows a higher adsorption capacity for HA compared with conventional adsorbents. The value of *k*_2_ for KOH-Hg (5.4 × 10^–2^ g mg^–1^ h^−1^) was higher than that for DW-Hg (4.7 × 10^–2^ g mg^–1^ h^−1^). HA has a zeta potential of approximately –35 mV at pH = 7. In the case of KOH-Hg, an increase in the number of electrostatic interactions with HA leads to an increase of the adsorption rate constant. To determine the material surface covered with adsorbed HA, we attempted to evaluate surface coverage (Sc) using the following equation [19]:

3.3 $$\text{Sc}=\frac{A_{\mathrm{HS}}N_{A}W}{M_{W}}\times \frac{1}{A_{\mathrm{ad}}}\times 100,$$

where *A*_HS_ is area per molecule, *N*_A_ is Avogadro's number, *W* and *M*_w_ are the adsorbed weight and molecular weight of HA, respectively, and *A*_ad_ is surface area of the adsorbent. The molecular size and weight of HA are 17 000 g mol^–1^ and 63 Å^2^, respectively [20]. The surface coverage of HA on KOH-Hg and DW-Hg after 24 h of the adsorption test were determined to be 76% and 66%, respectively. This implies that hydrogarnet has a great potential for HA adsorption even after the adsorption tests.

Fulvic acid (FA) is also one of the natural organic polyelectrolytes. FA has lower average molecular weight and functional group content than HA [21]. FA adsorption tests were carried out with an initial FA concentration of 30 ppm and 2.5 g l^–1^ adsorbent. The amount of FA adsorbed to both samples was found to be less than that of HA. The surface coverage of FA on KOH-Hg and DW-Hg after 24 h of the adsorption test were determined to be 20% and 15%, respectively. The molecular size and weight of FA is 570 g mol^–1^ and 86 Å^2^, respectively [22]. That is, hydrogarnet is a suitable adsorbent for HA.

To determine the influence of the changes to the chemical composition of hydrogarnet on its HA removal capacity, adsorption experiments were performed at hydrogarnet concentrations of 10, 15, 20, 25 and 30 mg l^−1^. Based on preliminary experiments, the equilibrium data fit the Freundlich equation (*R*^2 ^> 0.91) better than the Langmuir equation (*R*^2 ^> 0.88). Freundlich plots for the samples were described using

3.4 $$\ln\;q_{e}=\ln\;K_{F}+\frac{1}{n}\text{ln}\;C_{e},$$

where *q*_e_ (mg g^−1^) indicates the amount of HA adsorbed at equilibrium, *C*_e_ is the concentration of HA in solution at equilibrium (mg l^−1^), and *K*_F_ and *n* are the Freundlich constants which relate to the adsorption capacity and the energy for adsorption, respectively, and were calculated from the slope and intercept of the linear plots, as listed in table 1. *K*_F_ of KOH-Hg (3.26) is larger than that of DW-Hg (2.00), which shows a similar trend to that from the kinetic analysis. This suggests that the number of hydroxyl groups and different crystal structures affect the affinity for HA adsorption to the hydrogarnet. KOH-Hg has a higher value (0.87) for *n* compared with Hg (0.70). An increase of *n* was more homogeneous with respect to sorption in previous literature [23]. We suppose that KOH-Hg has various interaction sites for HA with various functional groups due to tuning of the hydroxyl groups and their crystal structures by changing their chemical compositions, resulting in an increase in the relative degree of homogeneity for HA adsorption compared with DW-Hg.

**Table 1.** Freundlich constants for HA adsorption to DW-Hg and KOH-Hg.

sample	*K*_F_	*n*
DW-Hg	2.00	0.70
KOH-Hg	3.26	0.87

E2, E3, E4 and E6 indicate absorbances at 250, 365, 465 and 665 nm, respectively. E2/E3 and E4/E6 ratios, which are bulk spectroscopic parameters, are widely used to evaluate the properties of HA including molecular weight, polarity and aromaticity [24]. A lower E2/E3 ratio originates from the higher average molecular weight and aromaticity of HA, while a decrease in the E4/E6 ratio is related to a decrease of polarity. The supernatant after 24 h of adsorption was used for evaluation by UV–Vis spectroscopy. Table 2 shows E2/E3 and E4/E6 ratios for HA and supernatant samples after adsorption testing. The E2/E3 ratio of the supernatant using KOH-Hg was higher than that using DW-Hg, while the supernatant using KOH-Hg had a lower E4/E6 ratio than that using DW-Hg. These results suggest that hydrogarnet is apt to adsorb material with higher average molecular weight and polarity (such as aromatic groups) in HA, indicating adsorption via hydrogen bonding at the surface (table 2). Si-OH and Al-OH groups in hydrogarnet act as adsorption sites for HA, resulting in enhancement of the adsorption capacity by tuning of these groups.

**Table 2.** E2/E3 and E4/E6 ratios for supernatant after adsorption tests using samples and HA.

sample	E2/E3	E4/E6
DW-Hg	0.96	1.21
KOH-Hg	0.86	0.46
HA	0.46	3.40

## 4. Conclusion

The HA adsorption properties of hydrogarnet were investigated to clarify the effects of hydroxyl group tuning on its adsorption. Controlling the chemical composition of hydrogarnet contributed to an increase in the number and development of diverse binding energies of these hydroxyl groups. Complex chemical compositions of the hydrogarnet strongly contributed to the adsorption properties, which were better in comparison with kaolinite and zeolite. The number of hydroxyl groups and their binding energies at the surface play an important role in enhancing its adsorption properties for HA.

## Supplementary Material

Data for the manuscript1.pptx

## Data accessibility

The datasets supporting this article have been uploading as part of the electronic supplementary material.
