# Correction to: ‘Adsorption behaviour of hydrogarnet for humic acid’

**DOI:** 10.1098/rsos.190240

**Published:** 2019-02-27

**Authors:** Hirotaka Maeda, Yuichi Kurosaki, Masanobu Nakayama, Emile Hideki Ishida, Toshihiro Kasuga

*R. Soc. open sci.* **5**, 172023. (Published 1 April 2018). (doi:10.1098/rsos.172023)

The calculation results for surface coverage of HA on samples on page 4 of the published paper should be corrected as follows.

‘The surface coverage of HA on KOH-Hg and DW-Hg after 24 h of the adsorption test were determined to be 76 and 66%, respectively’ should read: ‘…the adsorption test were determined to be 0.8 and 0.7%, respectively’.

