# Supplementary figures and images for: Adsorption behaviour of hydrogarnet for humic acid
Source: R Soc Open Sci. 2018 Apr 11;5(4):172023. doi: 10.1098/rsos.172023 (PMC5936920; doi:10.1098/rsos.172023)

## Slide 1
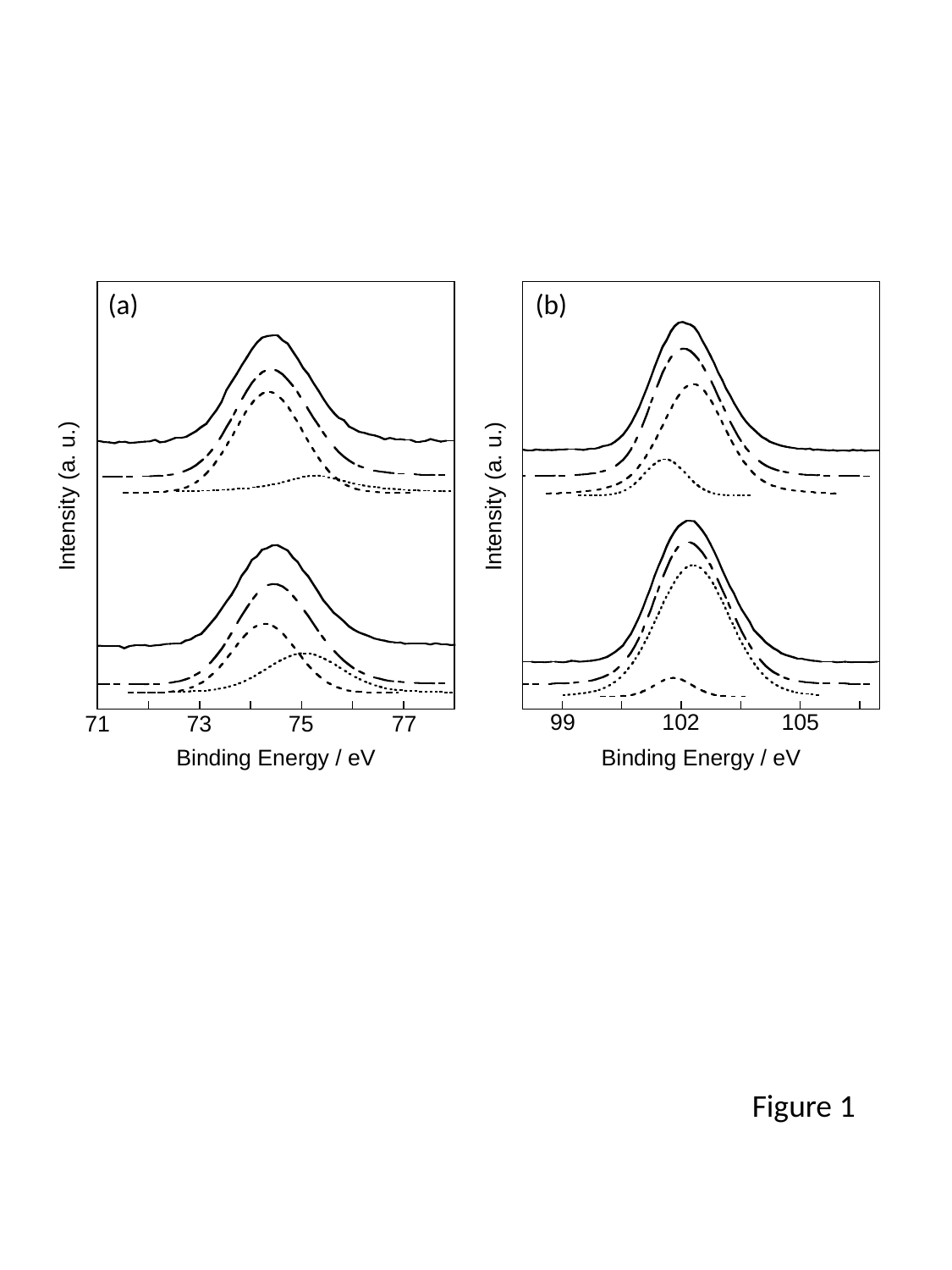

(a)
(b)
Figure 1

## Slide 2
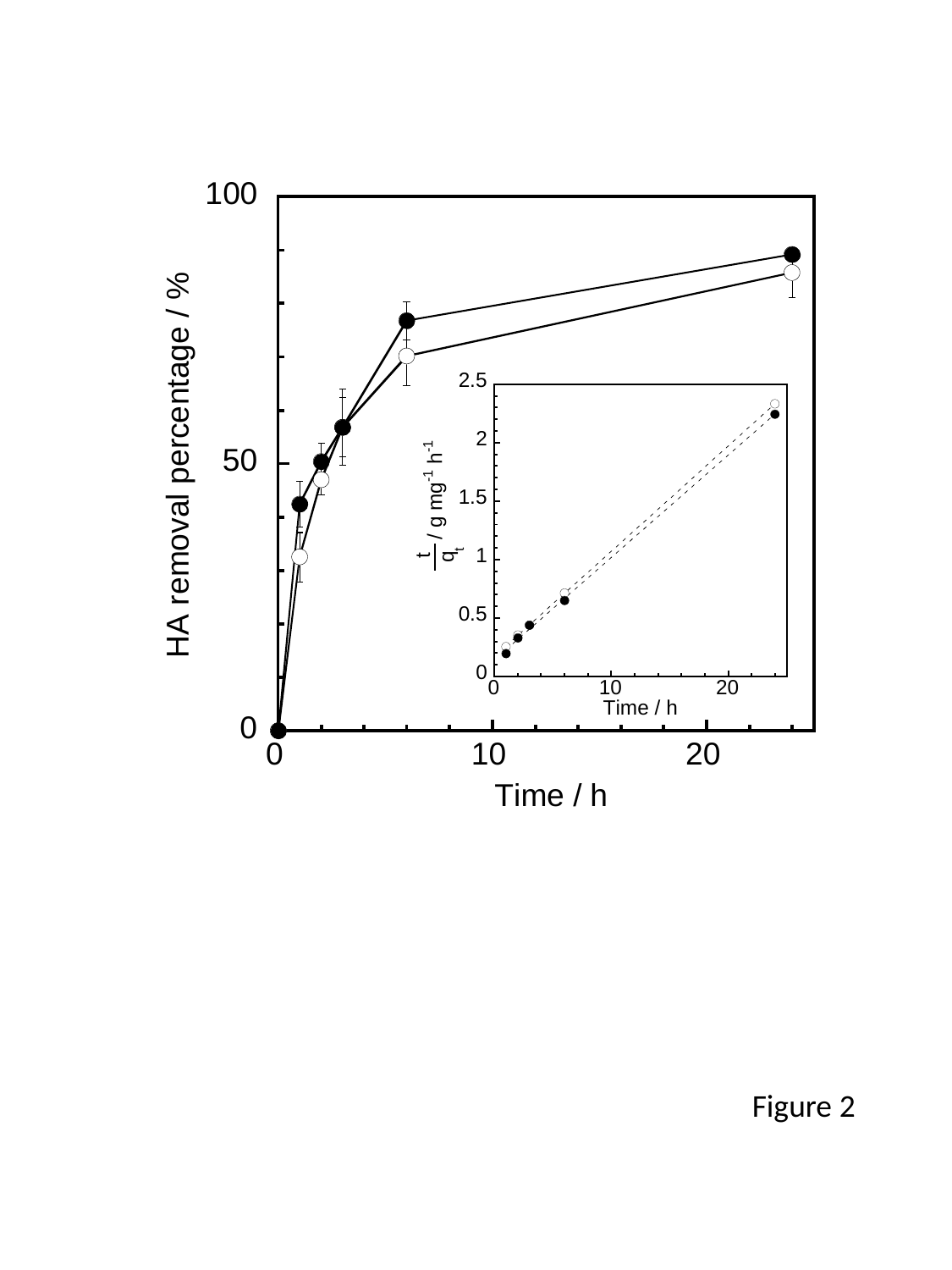

Figure 2

Supplement: Data for the manuscript1.pptx [file rsos172023supp1.pptx]
